# First principles study of surface properties and oxygen adsorption on the surface of Al_3_Ti intermetallic alloys

**DOI:** 10.1039/c8ra09175g

**Published:** 2019-01-14

**Authors:** Yang Zhou, Huihui Xiong, Yanhong Yin, Shengwen Zhong

**Affiliations:** Engineering Research Institute Jiangxi University of Science and Technology 86 Hongqi Road Ganzhou 341000 PR China yangzhou1998@126.com; Key Laboratory of Power Batteries & Relative Materials Ganzhou 341000 PR China zhongshw@126.com

## Abstract

The density functional theory (DFT) method was applied to study the structural, electronic and surface properties of low-index Al_3_Ti intermetallic materials. The surface energies and electronic structures of those surfaces were also discussed in this study. The calculated surface energies of the low-index surfaces of Al_3_Ti indicated that nonstoichiometric (110) surface with Al termination was the most stable surface. On this basis, the oxygen adsorption behavior of the (110)-Al surface was further studied to clarify the antioxidant mechanism of Al_3_Ti intermetallic alloys. Various adsorption sites of oxygen atoms on the (110)-Al surface were considered to identify the most stable adsorption configurations. According to the calculation results of adsorption energies, it was found that stability was maximized when oxygen was adsorbed at the Al–Al bridge site. Meanwhile, a density of state study indicated that adsorption of oxygen on the (110)-Al surface preferred to bond with Al atoms rather than Ti atoms.

## Abstract 2

The density functional theory (DFT) method was applied to study the structural, electronic and surface properties of low-index Al_3_Ti intermetallic materials.

## Graphical abstract

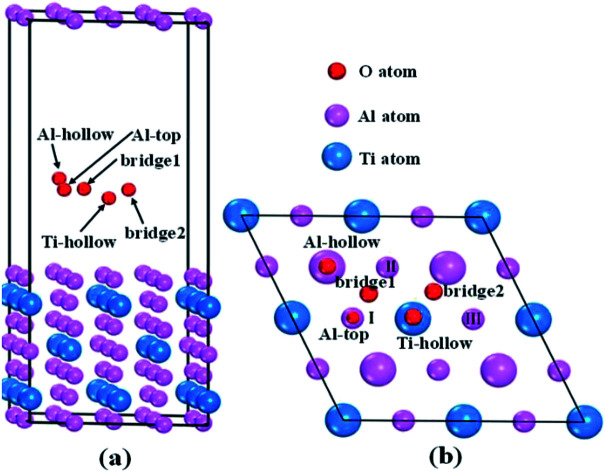

## 1. Introduction

Ti–Al intermetallic alloys are high temperature resistant structural materials with great potential in both aerospace and automotive applications and have been attracting particular attention.^1–4^ Compared with other two intermetallic alloys, namely, TiAl and Ti_3_Al, the Al_3_Ti alloy has the lowest density, largest elastic modulus, moderately high melting temperature and superior oxidation resistance.^5,6^ The oxidation and corrosion behavior of Al_3_Ti-based intermetallic compounds has been extensively investigated *via* experiments using high temperature conditions.^7–12^ Chen^11^ reported that Al–Ti alloys with an Al content of 50% shows low corrosion resistance at high temperatures, and the formation of mixed TiO_2_ and Al_2_O_3_ layers further decreased the corrosion resistance. Meanwhile, Parfitt^12^ studied the high-temperature oxidation behavior of Al_3_Ti intermetallic alloys in an oxygenated atmosphere and found that an α-Al_2_O_3_ layer formed on the alloy surface in the temperature range of 700–1200 °C, and this protective layer suppressed oxygen diffusion and slowed the oxidation rate. Recently, attempts had been made to further improve the oxidation resistance of Al_3_Ti alloy through alloying with Cr,^13,14^ Mn,^12^ Zr,^15^*etc.* For example, an investigation by Yamaguchi^16^ showed that Cu-substituted Al_3_Ti alloys exhibit very low oxidation resistance, while Mn, Ag, Fe, Cr-substituted alloys exhibited good oxidation resistance. All the studies showed that the oxidation behaviors of the Al_3_Ti alloys depended on its surface properties. Therefore, it was crucial to clarify the adsorption, dissolution, and diffusion properties of oxygen on the surface of Al_3_Ti alloy, which can reveal the mechanism of its oxidation. However, investigating the high-temperature corrosion of the alloys was challenging and could not be achieved by conventional methods.

First principles computations provided a suitable means to shed light on the physical and chemical surface properties. The adsorption of atomic and molecular oxygen on the surface of several Ti–Al intermetallic alloys such as γ-TiAl,^17,18^ Ti_3_Al,^19–21^ and the TiAl(111)/Al_2_O_3_(0001) interface^22^ were investigated theoretically using density functional theory (DFT) calculations. Liu^23^ used the DFT method to study the effect of surface self-segregation on the adsorption of oxygen by the γ-TiAl(111) surface, showing that Al self-segregation at the surface can enhance the interaction between O and Al atoms. Kulkova^24^ employed DFT calculations to explore the adsorption and diffusion of oxygen on γ-TiAl(001) and (100) surfaces, revealing the oxidation mechanism of γ-TiAl alloys. In this paper, the structural, electronic and surface properties of low-index surfaces of Al_3_Ti intermetallic alloy were studied by using the CASTEP code, which is based on the density functional theory and the periodical slab model. The most favorable adsorption site of oxygen atoms was determined by calculating the adsorption energies. According to the calculated state of density analysis, a bonding mechanism for the oxygen atoms and Al atoms was also presented, which was consistent with the experimental results.

## 2. Calculation method and details

All calculations in this study were performed using the Cambridge Serial Total Energy Package (CASTEP) code,^25,26^ which is based on the density functional theory. The interactions between the ionic core and valence electrons was modeled *via* the plane-wave ultra-soft pseudopotential method.^27^ The valence electrons of the atoms chosen were Ti 3s^2^3p^6^3d^2^4s^2^ and Al 3s^2^3p^1^. A generalized gradient approximation (GGA) of the Perdew–Burke–Ernzerhof (PBE) functional^28^ was employed to treat the exchange–correlation interactions. The Brillouin zone was sampled with the Monkhorst–Pack *k*-point grid. The cutoff energy and *k*-point sampling were set as 380 eV and 7 × 7 × 3 for the bulk, and 3 × 3 × 1 for all slabs, respectively.

**Fig. 1:**
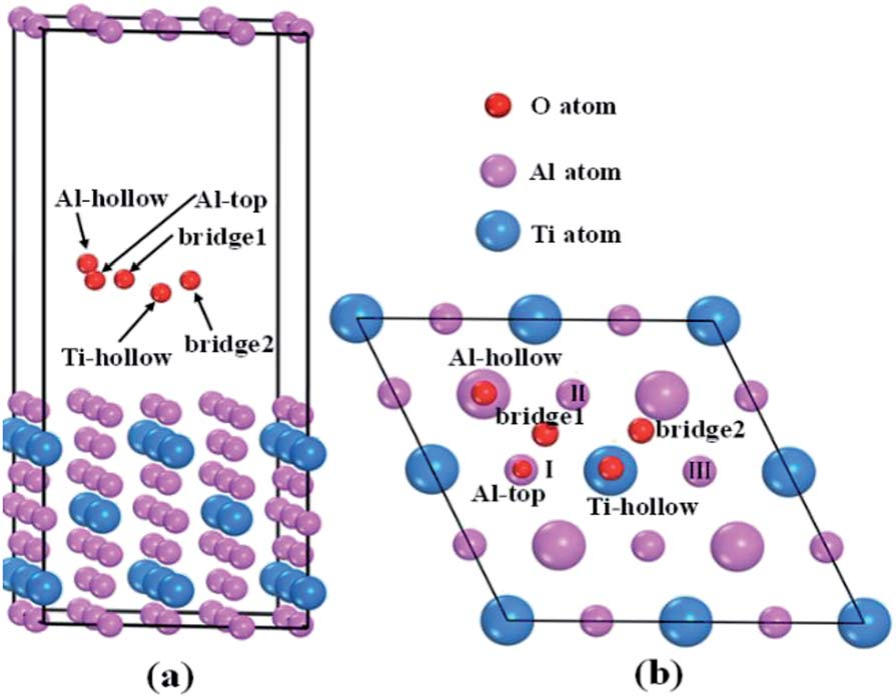
Schematic structure of the possible oxygen adsorption sites on an Al-terminated (110) surface: (a) side view, (b) top view. In (b), the small pink balls represent Al atoms in the surface layer, and the big pink and blue balls represent Al and Ti atoms in the subsurface layer, respectively.

To reveal the interaction mechanisms between oxygen and Al_3_Ti, the adsorption behavior of the oxygen atom was studied with different sites on the most stable surface of Al_3_Ti ((110)-Al surface, see Section 3.3). Fig. 1a shows the slab model of oxygen adsorption on the (110)-Al surface, which consisted of seven layers containing 56 atoms and separated with 15 Å spacing of vacuum. The atoms located on the three bottom layers of the slab were fixed, while the atoms on the top-most four layers and oxygen atoms were allowed to relax. To prevent the mutual effect of the adsorbed oxygen atoms, the supercell of the (110)-Al surface model (Fig. 1b) was enlarged by (2 × 2) along the surface. The surfaces were fully relaxed before the oxygen adsorption calculations were performed.

The single oxygen atom was placed on five possible adsorption sites of the (110)-Al surface of Al_3_Ti. These sites included the Al-top site, the bridge1 site, the bridge2 site, the Al-hollow site, and the Ti-hollow site, which are defined by the location of the oxygen atom, respectively, as follows: at the top of Al, Al–Al(ii) in the surface layer, Al(ii)–Al(iii) in the surface layer, Al in the second surface layer, and Ti in the second surface layer. The adsorption energy (*E*_ads_) was calculated from the following equation:^29^

1 *E*_ads_ = *E*_O/Al_3_Ti(110)_ − *E*_Al_3_Ti(110)_ − *E*_O_

where *E*_O/Al_3_Ti(110)_ and *E*_Al_3_Ti(110)_ were the total energies of the (110)-Al surface with and without oxygen adsorption, respectively, and *E*_O_ was the energy of a free oxygen atom, which was calculated by placing an O atom into an empty cell of 10 × 10 × 10 Å. The spin polarization was considered during the calculation process and the obtained value was −431.79 eV. A negative adsorption energy indicated that the adsorption was thermopositive and the adsorption site was stable. Moreover, larger absolute values of adsorption energy indicated more stable adsorption site. The possible stable adsorption sites of oxygen atom at the top, bridge and hollow locations could be determined by optimizing the structural parameters.

## 3. Results and discussion

### 3.1 Bulk properties

To guarantee the accuracy of the calculations performed in this study, the lattice constant and formation enthalpy of Al_3_Ti were first calculated. The formation enthalpy (Δ*H*_Al_3_Ti_) of Al_3_Ti could be obtained using the following equation:

2 Δ*H*_Al_3_Ti_ = (*E*_Al_3_Ti_ − 3*E*_Al_ − *E*_Ti_)/4

where *E*_Al_3_Ti_ was the total energy of an Al_3_Ti unit cell, and *E*_Al_ and *E*_Ti_ were the energies of a single Ti and Al atom in the bulk state, respectively.

The calculated structural parameters and formation enthalpy of Al_3_Ti intermetallic alloy were shown in Table 1. The lattice constants “*a*” and “*c*” of bulk Al_3_Ti were 3.8501 Å and 8.6274 Å, which were in good agreement with the experimental results published in ref. 32 and 34, respectively. Additionally, the formation enthalpy obtained in our study was −153.82 kJ mol^−1^, which matched well with the other calculated and experimental results. Meanwhile, the negative value for the formation enthalpy of Al_3_Ti suggested that tetragonal Al_3_Ti was a stable state. Therefore, the calculation methods could guarantee accuracy and reliability of subsequent calculations.

**Table 1:** Calculated lattice parameters and formation enthalpy of Al_3_Ti along with previously reported theoretical and experimental data

Phase	Method	*a* (Å)	*c* (Å)	Formation enthalpy (kJ mol^−1^)
Al_3_Ti	This work	3.8501	8.6274	−153.82
	GGA-PBE^30^	3.868	8.628	−160.40
	GGA-PW91 (ref. 31)	3.851	8.611	—
	Expt.^32^	3.8537	8.5839	−151.20 (ref. 33)
	Expt.^34^	3.851	8.610	—

**Fig. 2:**
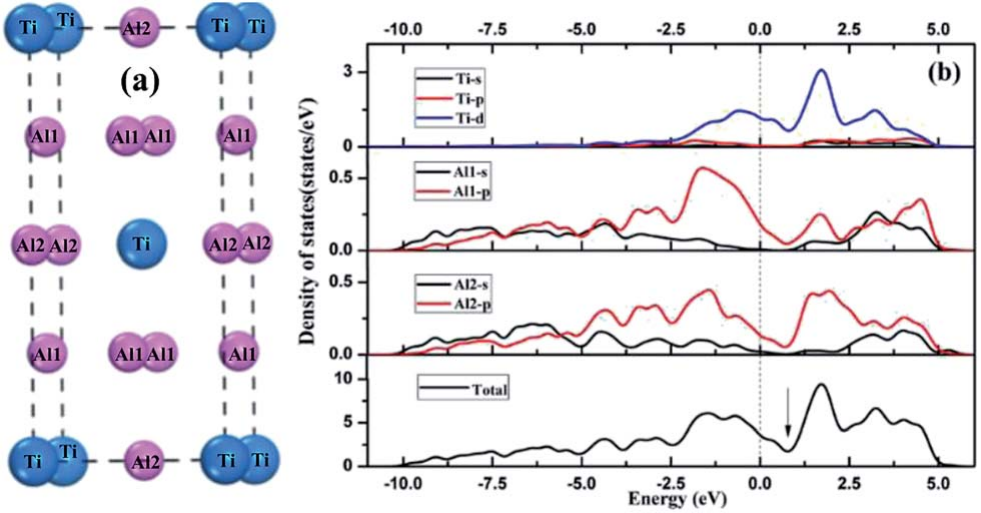
Crystal structures (a) and density of states (b) of bulk Al_3_Ti.

Tetragonal Al_3_Ti belonged to space group *I*4/*mmm*. The Ti, Al1 and Al2 atoms occupy (0, 0, 0), (0, 0, 0.5) and (0, 0.5, 0.25) Wyckoff sites, respectively. Fig. 2 presents the total and partial density of states (DOS) distributions of bulk Al_3_Ti. The contributions of Al1 and Al2 atoms to DOS differed slightly, which was probably due to the different occupied locations. The existence of metallic bonds in the Al_3_Ti intermetallic alloy was also proved by the DOS profile across the Fermi level. Moreover, the pseudogap near the Fermi level indicated the existence of covalent bonding,^35^ which was in accordance with the result that covalent interactions were possessed by the 3d orbit of Ti atom and 2p orbit of Al atom.^36^

### 3.2 Surface electronic properties

**Fig. 3:**
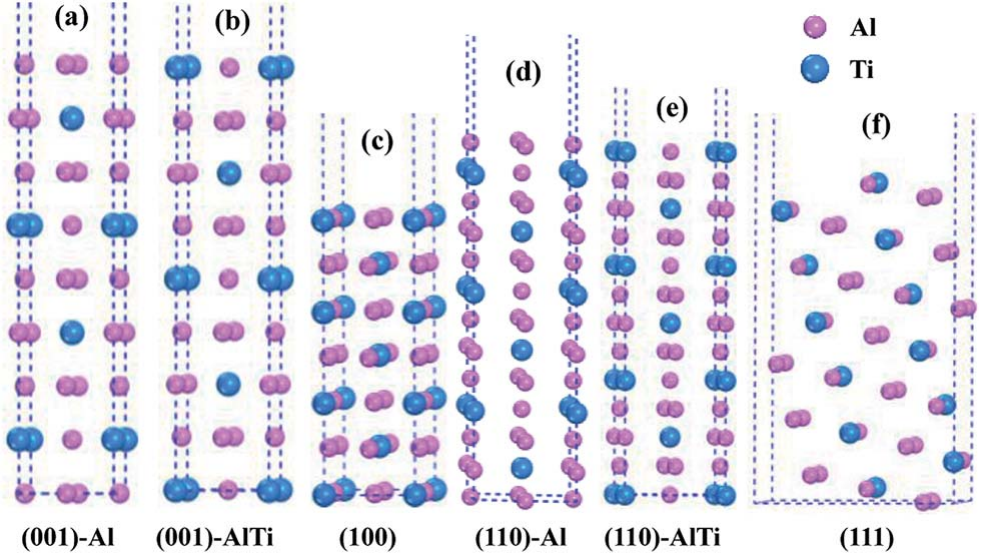
The constructed low-index surfaces of Al_3_Ti: (a) non-stoichiometric (001) surface with Al termination, (b) non-stoichiometric (001) surface with AlTi termination, (c) stoichiometric (100) surface, (d) non-stoichiometric (110) surface with Al termination, (e) non-stoichiometric (110) surface with AlTi termination and (f) stoichiometric (111) surface.

**Fig. 4:**
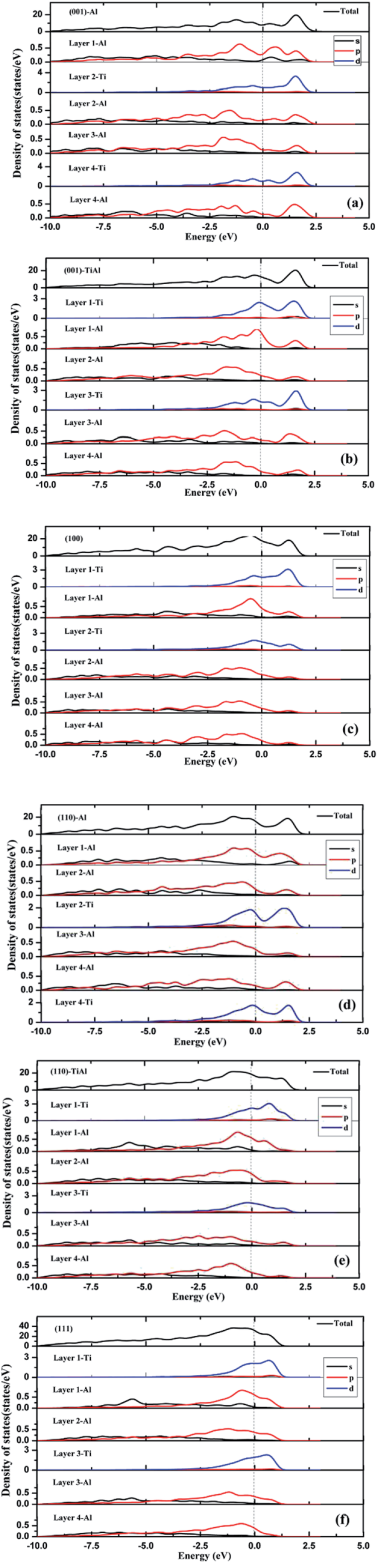
The TDOS and PDOS of various low-index surfaces: (a) (001)-Al, (b) (001)-AlTi, (c) (100), (d) (110)-Al, (e) (110)-AlTi, (f) (111).

As shown in Fig. 3, the low-index surfaces of Al_3_Ti were constructed as follows: nonstoichiometric surfaces with (a, d) Al termination and (b, e) AlTi termination, and (c, f) stoichiometric surfaces. The calculated partial density of states (PDOS) and total density of states (TDOS) are shown in Fig. 4. According to the calculated TDOS results, all of the low-index surfaces exhibited metallic properties and a pseudogap near the Fermi level, which is consistent with the DOS of bulk Al_3_Ti (Fig. 2). The PDOS of Ti and Al in the inner layers were also similar to those of bulk Al_3_Ti, but noticeable changes in the contours of the outermost layer occurred relative to the inner layer. For the nonstoichiometric (001) and (110) surfaces with AlTi termination (Fig. 4b and e), the surface Ti-3d and Al1-2p electrons moved towards the Fermi level, suggesting an increased metallic property and decreased stability. However, the Al-terminated surface (Fig. 4a and d) Al-2p electrons were far from the Fermi level, suggesting a decreased metallic property and increased stability. For the two stoichiometric surfaces (Fig. 4c and f), the non-localization of the electrons in the surfaces was in the order of (100) > (111) on the basis of the intensity of the DOS, which indicated that the (111) surface was more stable than the (110) surface.

### 3.3 Surface energy

The surface energy (*γ*_s_) was used as a basic parameter to compare the stability of various low-index surfaces, which was calculated from the following equation:^37–39^3
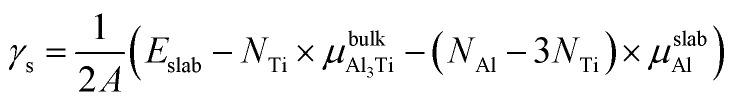
where *E*_slab_ refers to the total energy of the relaxed surface; *N*_Ti_ and *N*_Al_ are the numbers of the Ti and Al atoms in the slab, respectively; 
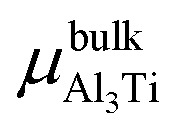
 and *μ*^slab^_Al_ are the chemical potentials for bulk Al_3_Ti and Al atoms in surface slab, respectively; and *A* is the surface area. The chemical potential of Al was smaller than that of the corresponding bulk substances. Moreover, the chemical potential of Al was related to the chemical potential of bulk Al_3_Ti, which was defined as:4
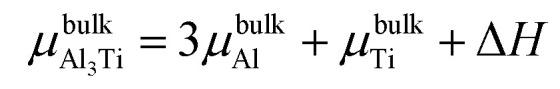
5
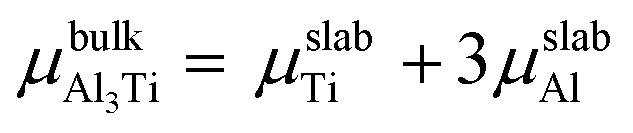
where *μ*^bulk^_Al_ and *μ*^bulk^_Ti_ are the single atomic energy of bulk Al and bulk Ti, respectively; Δ*H* is the formation enthalpy of Al_3_Ti; and 
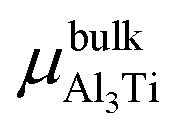
 is the total energy of a unit cell. According to eqn (4) and (5),

6 *μ*^bulk^_Ti_ − *μ*^slab^_Ti_ + Δ*H* = 3(*μ*^slab^_Al_ − *μ*^bulk^_Al_)

In view of the negative value of the formation enthalpy of bulk TiAl_3_ (the calculated value in this work was −1.59 eV per unit cell), the range of Al chemical potential (Δ*μ*_Al_) was:7
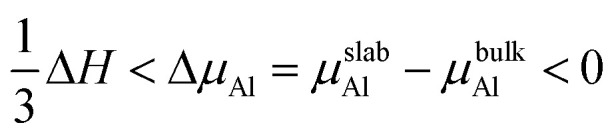


**Fig. 5:**
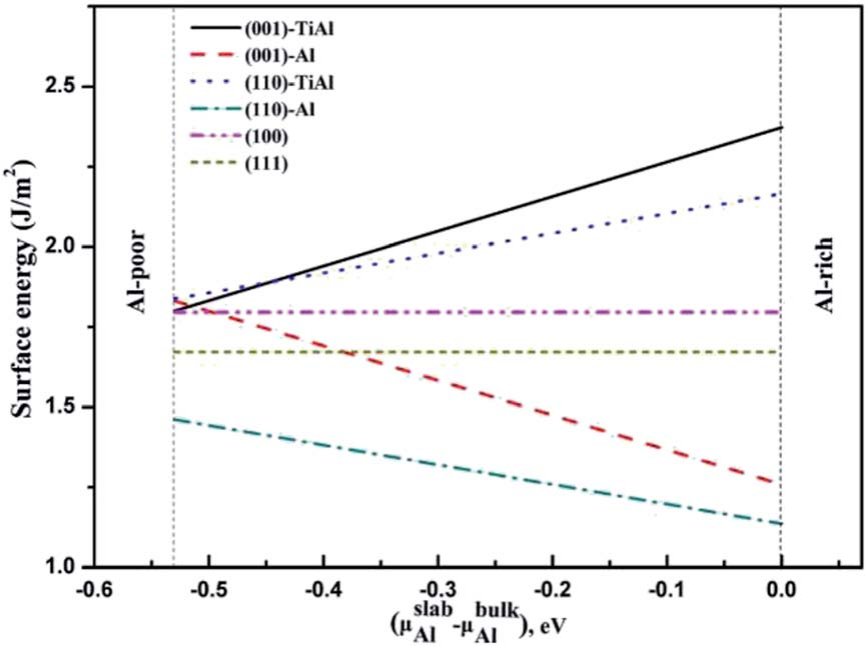
Relationship between surface energies of low-index surfaces and Al chemical potential.

The surface energies of the low-index surfaces were calculated on the basis of the above formulas. As shown in Fig. 5, the surface energies of (100) and (111) do not depend on the chemical potential of Al due to their stoichiometric surfaces. However, the surface energies of the nonstoichiometric surfaces such as TiAl-terminated surfaces increased with the chemical potential of Al, but the surface energies of the Al-terminated surfaces inversely decreased. The stability under Al-rich conditions was in the order of: (110)-Al > (001)-Al > (111) > (100) > (110)-AlTi > (001)-AlTi; under Al deficient conditions, the stability decreased in the order of: (110)-Al > (111) > (001)-Al > (001)-AlTi > (110)-AlTi. In all, the (110)-Al surface was stable under a wide range of Al chemical potentials.

### 3.4 O adsorption on the (110)-Al surface

**Fig. 6:**
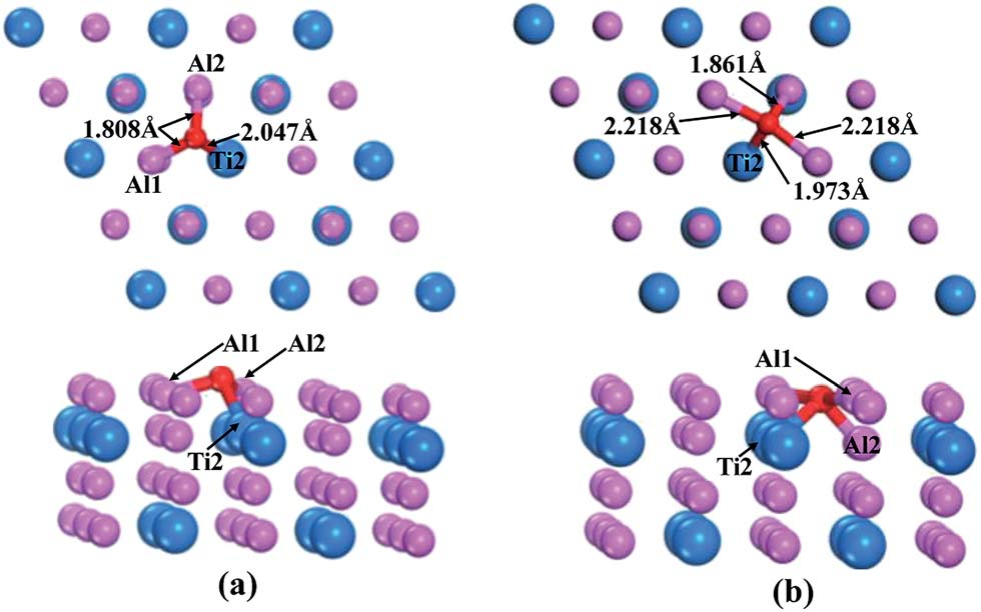
Top and side views of O adsorption on bridge1 (a) and bridge2 (b) sites after geometrical optimization.

According to the above calculations, the (110)-Al surface of Al_3_Ti was the most stable; thus, the behavior of oxygen atoms adsorbed on the surface were investigated to further reveal the oxygenation process. The calculated adsorption energies of oxygen at different sites on the (110)-Al surface are listed in Table 2. The adsorption site had a strong effect on the adsorption energy. The negative adsorption energies were indicative of a spontaneous adsorptions process. Moreover, the *E*_ads_ of the O atoms at bridge sites were larger than those at Ti-hollow, Al-hollow and Al-top sites, suggesting that O atoms preferentially adsorbed at the bridge positions. According to the comparison of adsorption energies, it could be concluded that the adsorption of O on the bridge1 sites was more stable than on the bridge2 sites. The initial distances between the O atoms and the nearest neighboring metal atom were about 3.5 Å. After relaxation, the lengths of the O–Al1 bond for the bridge1 and bridge2 sites were 1.808 Å and 2.218 Å (Fig. 6a and b), respectively, which corresponds to the O–M bond lengths in metal oxides. Therefore, the adsorption of oxygen might result in the formation of the corresponding oxides. In addition, when oxygen atom was adsorbed on bridge2 position, the bonding strength of O–Al2 (1.861 Å) was stronger than that of O–Al1 (2.218 Å) (Fig. 6b), which indicated that the O atoms preferentially moved to the interstitial site.

**Table 2:** Adsorption energy of oxygen at different sites of the (110)-Al surface

Surface	Site	*E*_ads_ (eV)
(110)-Al	Ti-hollow	−5.17
	Al-hollow	−4.53
	Al-top	−4.00
	bridge1	−7.13
	bridge2	−6.48

**Fig. 7:**
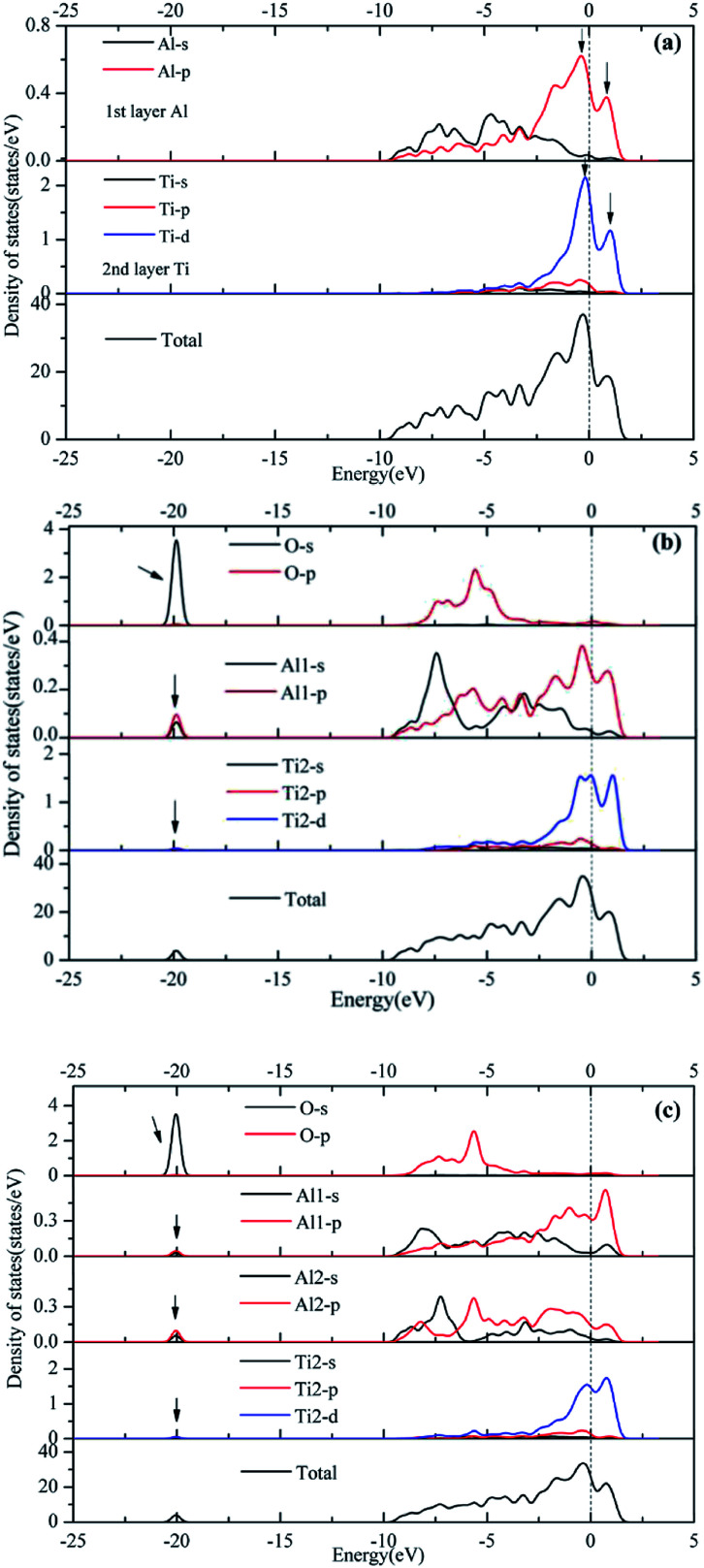
Partial density of state (PDOS) and total density of state (TDOS) analyses of (a) free surface, (b) oxygen at the bridge1 site, (c) oxygen at the bridge2 site.

Orbital hybridization and bonding characteristics were further analyzed according to the total and partial densities of states without and with O adsorption on the bridge sites (Fig. 7). For the free (110)-Al surface (Fig. 7a), two significant peaks that formed in the vicinity of Fermi energy led to strong metallic bonding. Moreover, the surface TDOS was mainly contributed from the interactions between Al-p and Ti-d orbitals. When an O atom was adsorbed on the bridge1 site (Fig. 7b), the peak height of the DOS of both the Al and Ti atoms decreased compared with those in the free surface sites, which suggested that the partial charges of the Al and Ti atoms were transferred to the O atom in the formation of strong covalent bonds. Meanwhile, clear hybridizations between Al-sp and Ti-d orbitals occurred in the range of −10 to 2.5 eV. Additionally, weak interactions could also be observed between O-s, Al-s, Al-p and Ti-d orbitals around −20 eV due to emergence of several new peaks for O, Al and Ti, and the region located around −20 eV was mainly dominated by O-s orbitals. Similar characteristics could also be found on the bridge2 site (Fig. 7c). The Mulliken charges and bond populations for O adsorption on the bridge1 and bridge2 sites are listed in Table 3. The Al1, Al2 and Ti2 in the two adsorption systems are defined in Fig. 6. The charge transfers between the metal and O was the most obvious feature of the Mulliken charges in both the adsorption systems. The charge of the Al2 atom was larger than that of Ti2, which indicated that the Al atom exhibits greater electron loss than Ti. Furthermore, the bond population of O–Al2 was larger than that of O–Ti2, while the bond length of O–Al2 was shorter than that of O–Ti2. This indicates that the O atom preferentially bonds with an Al atom rather than a Ti atom. Meanwhile, the available experimental data for the high-temperature oxidation of the Al_3_Ti alloy in an oxygenated atmosphere^40,41^ has shown the formation of an outer protective oxide layer that almost completely consists of α-Al_2_O_3_; this layer led to a suppressed oxygen diffusion, which significantly reduced the oxidation rate of the alloy. The theoretical and experimental results agreed with each other.

**Table 3:** Mulliken charges, bond lengths (Å) and populations of the bridge1 and bridge2 adsorption systems

Adsorption site	Atom	s	p	d	Total	Net *e*	Bond type	Bond length	Population
bridge1	O	1.88	5.03	0	6.91	−0.91	O–Al1	1.808	0.41
	Al1	1.08	1.45	0	2.54	0.46	O–Al2	1.808	0.41
	Al2	1.08	1.45	0	2.54	0.46	O–Ti2	2.047	0.30
	Ti2	2.30	6.55	2.80	11.64	0.36			
bridge2	O	1.88	4.96	0	6.84	−0.84	O–Al1	2.218	0.16
	Al1	1.25	1.53	0	2.87	0.22	O–Al2	1.861	0.34
	Al2	1.01	1.66	0	2.67	0.33	O–Ti2	1.973	0.32
	Ti2	2.30	3.55	2.83	11.68	0.32			

## 4. Conclusions

In summary, the structural, electronic and surface properties of the Al_3_Ti intermetallic alloy were investigated by the DFT method. The calculated bulk properties were in good agreement with the available experimental results. According to the density of states and surface energies, a nonstoichiometric (110) surface with Al termination was found to be the most stable surface. Strong hybridizations between O-p, Al-p and Ti-d orbitals contributed to the adsorption behavior of oxygen on the (110)-Al surface. The O atoms showed a preference for adsorption at the bridge1 site based on the lowest adsorption energy. Density of state analysis revealed that the interaction between the O-2p and Al-2p orbitals is relatively strong, which promotes the formation of oxides like Al_2_O_3_ instead of pure protective alumina and negatively influences the oxidation resistance of Al_3_Ti intermetallic alloy.

## Conflicts of interest

There are no conflicts to declare.

## Supplementary Material
